# Dyadic Agreement in the Perceptions of Patient Disability Between the Stroke Patient and Rehabilitation Therapist

**DOI:** 10.1093/geroni/igaa057.3353

**Published:** 2020-12-16

**Authors:** Naoki Takashi Michael McCarthy, Rie Suzuki, Kakuya Ogahara, Masako Kihara, Masahiro Kihara, Takeo Nakayama

**Affiliations:** 1 Northern Arizona University, Flagstaff, Arizona, United States; 2 University of Michigan-Flint, Flint, Michigan, United States; 3 Kanagawa University of Human Services, Yokosuka city, Kanagawa, Japan; 4 Kyoto University, Kyoto City, Kyoto, Japan

## Abstract

Research supports that an agreement about the consequences of the illness within related parties is critical for optimal patient outcomes. This study aimed to explore the association between patient QOL and the degree of agreement in the perceptions of patient disability within the stroke patient-rehabilitation therapist dyad. A cross-sectional study was conducted in Japan from March 2019 to February 2020. A total of 81 dyads consisting of a male stroke patient living at home and the therapist in charge of the eligible patient participated. Patient QOL was measured using the WHOQOL BREF. Perceptions of patient disability were measured using the 12-item WHO Disability Assessment Schedule 2.0 (DAS). DAS scores of patients and therapists were classified into two (high, low) and three (high, medium, low) categories, respectively, and six patterns of agreement on patient ability were created to use in the analysis. Generalized estimating equations were used to examine multivariable associations between the degree of agreement within dyad and WHOQOL scores in patients. Results suggested that when the patient appraised himself as having a low disability, the degree of patient-therapist disagreement was negatively associated with patient QOL. When the patient appraised himself as having a high disability, his QOL was lower, regardless of the degree of agreement. Disagreements in the perception of disability between patients and therapists can worsen patient QOL, especially when the patient perceives himself as having a low disability.

